# Supplementary material for: Microglia morphological response to mesenchymal stromal cell extracellular vesicles demonstrates EV therapeutic potential for modulating neuroinflammation
Source: J Biol Eng. 2024 Oct 17;18:58. doi: 10.1186/s13036-024-00449-w (PMC11488223; doi:10.1186/s13036-024-00449-w)
Supplement: Supplementary file 6 — Supplementary Material 6 [file 13036_2024_449_MOESM6_ESM.docx]

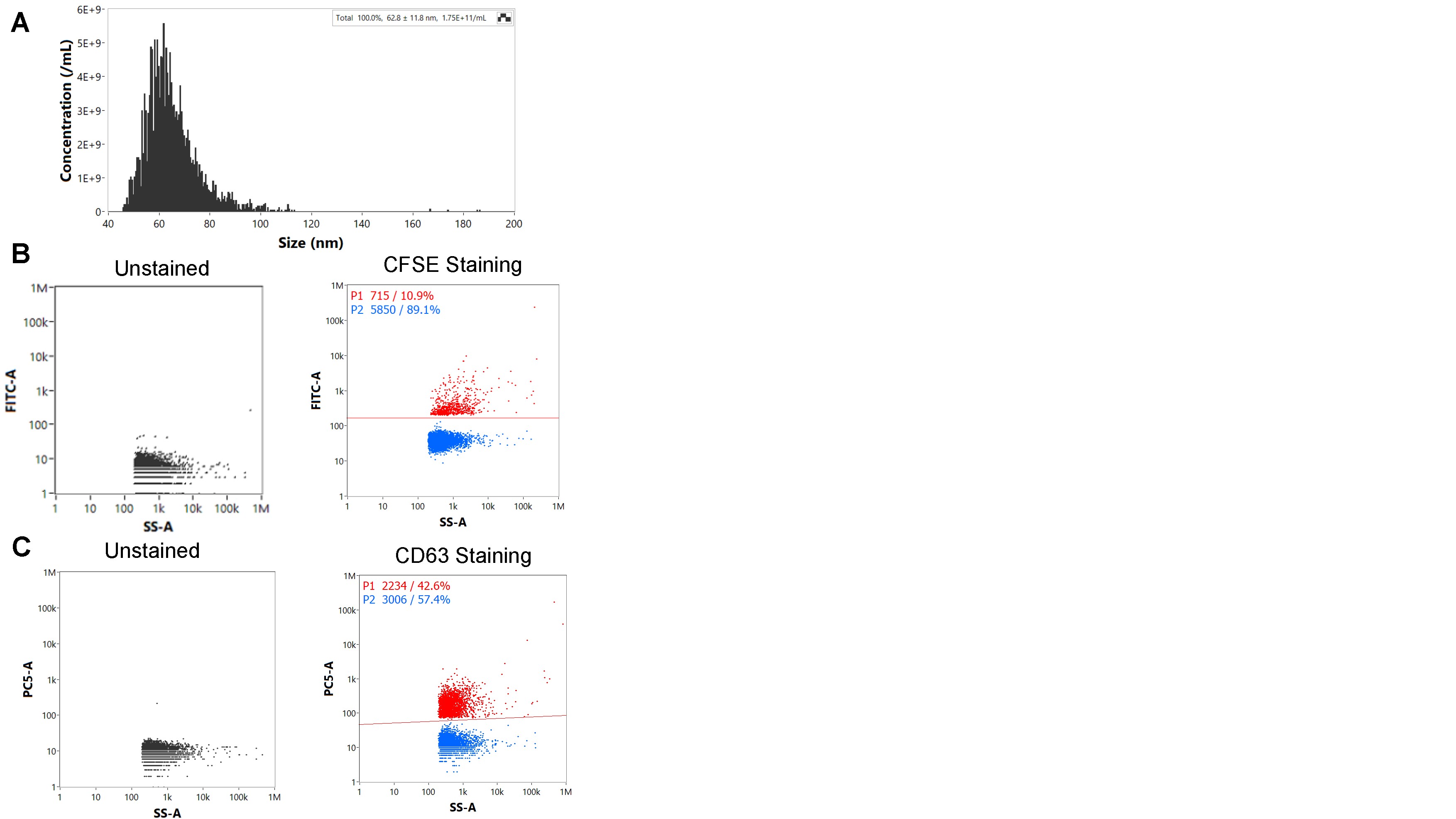


**Figure S1: MSC-EV surface marker staining  (A)** Concentration (particles/mL) and Size distribution (nm) of isolated EVs. Gating Strategy for single stains namely CFSE **(B)** and CD63 **(C)** conducted independently**.**

**Figure S2: Microglia morphology changes upon activation with cytokines across multiple experiments consistently.** Principal component 1 (PC1_morpho_) calculated using 21 features represents 51.1% of the variance between –CTL and +CTL. Each point is a mean 6 wells containing ~700-1000 cells/well with mean and standard deviation plotted for n=5 experiments per group. *p<0.01 vs -CTL for all groups conducted using paired t-test (two-tailed). Each experiment is represented by a different symbol.

**Figure S3: Stimulated microglia morphology changes upon treatment with MSC-EVs from multiple batches.** Normalized principal component 1(PC1_morpho_) calculated using 21 min-max normalized features represents 51.2% of the variance. Each point is a mean of 6 wells calculated using median of ~700-1000 cells/well for n=5 experiments. *p<0.05 vs +CTL for all groups conducted using RM one-way ANOVA with multiple corrections using Dunnett multiple comparison testing.

 **Figure S4: Dose-dependent response of microglia to stimulation and MSC-EVs.** Microglia morphological response over 4 different concentrations of IFN-γ & TNF-α (0.4/5/25/50 ng/mL for each cytokine). Principal component 1(PC1_morpho_) calculated using 21 features represents 48.5% of the variance. Each point is a mean of 4-6 wells calculated using median of ~700-1000 cells/well. *p<0.05 vs –CTL, #p<0.05 vs +CTL (within the respective IFN-γ/TNF-α treatment) for all groups conducted using Brown-Forsythe and Welch one-way ANOVA tests with multiple corrections using Dunnett T3 testing.

​

 **Figure S5: Micorglia respond to MSC-EV treatment in a dose dependent manner.** Microglia morphological response upon stimulation (IFN-γ & TNF-α, 5ng/mL, +CTL) and EV treatment (1X,16X; where X is the dilution factor). Principal component 1 (PC1_morpho_) calculated using 21 features represents 74% of the variance. Each point is a mean of 8 wells calculated using median of ~700-1000 cells/well. *p<0.05 vs –CTL, #p<0.05 vs +CTL, @p<0.05 vs EVs (1X) for all groups conducted using Brown-Forsythe and Welch one-way ANOVA tests with multiple corrections using Dunnett T3 testing.

​

**Figure S6: PCA Plot of unannotated lipids.** Positive and Negative mode PCA plots of all lipids identified across the experimental groups –CTL, +CTL and +EVs with n=3 technical replicates. ​
